# Proteomics Profiling of Chimeric-Truncated Tissue Plasminogen activator Producing- Chinese Hamster Ovary Cells Cultivated in a Chemically Defined Medium Supplemented with Protein Hydrolysates

**DOI:** 10.18869/acadpub.ibj.21.3.154

**Published:** 2017-05

**Authors:** Bahareh Azarian, Seyedeh Matin Sajedin, Amin Azimi, Mozhgan Raigani, Behrouz Vaziri, Fatemeh Davami

**Affiliations:** 1 Protein Chemistry Unit, Biotechnology Research Center, Pasteur Institute of Iran, Tehran, Iran; 2 Department of Microbiology, Science and Research Branch, Islamic Azad University, Guilan, Iran; 3 Department of Biotechnology, College of Science, University of Tehran, Tehran, Iran; 4 Eukaryotic Expression Unit, Biotechnology Research Center, Pasteur Institute of Iran, Tehran, Iran

**Keywords:** CHO cells, Hydrolysates, Proteomics

## Abstract

**Background:**

Culture media enrichment through the addition of protein hydrolysates is beneficial for achieving higher protein expression.

**Methods:**

In this study, designing the optimum mixture of four soy and casein-derived hydrolysates was successfully performed by design of experiment and specific productivity increased in all predicted combinations. Protein profile of recombinant CHO (rCHO) cells producing tissue plasminogen activator in a serum-free medium (SFM) supplemented with designed hydrolysate additives was compared to that of rCHO cells cultivated in SFM.

**Results:**

Identification of differentially expressed proteins using two-dimensional gel electrophoresis coupled with MALDI-TOF/TOF revealed the role of energy metabolism related proteins and importance of prevention of oxidative stress by this special media enrichment strategy. Up-regulation of mitochondrial enzymes, pyruvate dehydrogenase E1, and Peroxiredoxin-III, as well as other proteins involved in metabolic pathways, and uridine monophosphate/cytidine monophosphate kinase indicated higher metabolic activity. Furthermore, along with antioxidant effect of peptones, proteins with antioxidant function such as ferritin and peroxiredoxin-III were up-regulated.

**Conclusion:**

Understanding molecular mechanisms involved in enhancement of protein expression can provide new approaches for efficiently engineering rCHO cell. These results support the competence of proteomics studies in finding new insights to biochemical pathways for a knowledge-based optimization of media compositions.

## INTRODUCTION

Chinese hamster ovary (CHO) cells are the most commonly used expression hosts in biopharmaceutical industry for production of recombinant proteins, especially monoclonal antibodies. Industrial applications of CHO cell have motivated researches to improve its expression characteristics, mainly higher specific productivity (q), through cell engineering or culture condition optimization[1-4].

Limitations in using animal-derived additives, due to the potential risk of infectious contaminants, make serum-free medium (SFM) a preferred choice. Non-animal (especially plant) derived additives are usually used as an effective additive to compensate the lower specific productivity of SFM-cultivated cells[5-7]. Our previous studies showed the positive effect of hydrolysates from plant and casein sources on volumetric productivity of CHO cells[3,4]. The increase of an Fc-fusion protein titer in CHO cells using plant-derived hydrolysates has been reported by Huang *et al*.[7]. Improving growth profile and recombinant protein production as a result of supplementation with the plant-derived hydrolysates has also been reported in other mammalian cells such as hybridoma, HEK, and BHK cells[8].

The culture media optimization process is labor-intensive and time-consuming as it requires manipulation of multiple factors, and each factor has many states or sometimes a continuous range, i.e. concentration.. An efficient tool to save time and make such studies easier and doable is the design of experiment (DOE) through which optimum condition(s) out of all available ones and the correctness of the predictions must be tested by experiments.

In this research, we tried to achieve a mixture of four protein hydrolysates, which would maximize the specific production of a novel chimeric-truncated form of tissue plasminogen activator (t-PA)[9-11] in CHO cells cultivated in SFM supplemented with DOE-designed optimized mixtures. We also investigated the proteins and molecular mechanisms involved in the enhancement of specific productivity by two-dimensional gel electrophoresis coupled with mass spectrometry technique. Previous proteomics studies on CHO cells producing recombinant protein were performed using the 2DE-MS technique to investigate the intracellular effect of supplemented mixture hydrolysates in SFM[8] and applying different concentration of sodium butyrate[12]. A recent study on non-gel-based techniques attempted to specify cellular mechanisms involved in protein expression[13].

## MATERIALS AND METHODS

### General materials

Serum-free CD DG44 medium, DG44 transfection kit, and antibiotic Zeocin were purchased from Invitrogen-Gibco (USA). TubeSpin^®^ bioreactors were obtained from Sartorius Stedium (Switzerland). The Chromolize t-PA Assay Kit was purchased from Biopool (Ireland), and goat anti-rabbit IgG-HRP conjugate was obtained from Santa Cruz biotechnology (CA, USA). The rabbit polyclonal antibody for t-PA was supplied from Abcam (MA, USA), and peptones from Organotechnie (La Courneuve, France). The specification of peptones, including total amino acid composition, molecular weight distribution, and free amino acid content was provided by the company (Table 1).

**Table 1:** Total amino acids content, average molecular weight (MW) and MW distribution of the peptones evaluated in this study

Name	Origin	Catalogue No.	Total amino acid content (g/100 g)	Average MW (daltons)		MW distribution (%)			

						<0.3 (kDa)	0.3-1 (kDa)	1-10 (kDa)	>10 (kDa)
Trypton N1	Casein	19553	81.6	490		31.7	60.1	8.2	0
Casein peptone plus	Casein	19544	85.1	491		38.5	53.0	8.5	0
Peptone E110	Soy	19885	49.4	1,206		31.1	48.7	18.5	1.9
Peptone A2SC	Soy	19649	53.8	503		30.6	60.8	8.6	0

### Cell culture

Suspension-adapted CHO-DG44 cells were seeded at a concentration of 2×10^5^ cells/mL in a serum-free CD DG44 medium with 8 mM glutamine at 37°C. The cells were incubated at 37°C for 10 days in 50-ml disposable TubeSpin^®^ bioreactors containing 10 mL CD DG44 medium supplemented with different ratios of hydrolysates. The disposable TubeSpins were shaken at 110 rpm on a orbital shaker with a shaking diameter of 5.0 cm placed in a 5% CO_2_ incubator and 95% humidity.

### Hydrolysate mixture optimization

The effect of peptone supplementation on growth profile and specific productivity of CHO-DG44 cells were investigated based on our previous studies[3,4]. To this end, four different sources of peptones, including casein peptone plus, Tryptone N1 from casein, Soy peptone A2 SC, and soy peptone E110, with the greatest effect in the CD DG44 basal media were selected. Total concentration of 2 and 5 gL^−1^ of hydrolysate additive was determined as optimum concentrations for productivity, toxicity, and viability using different concentrations of Soy peptone E110 (data not shown). Matrix of 20 mixtures of four hydrolysates were provided using a simplex lattice design, quadratic mixture model, by DOE software Design-Expert^®^ (version 6.0; Stat-Ease Inc., Minneapolis, MN, USA). SFM was supplemented with the hydrolysates with the final concentrations of 2 and 5 g L^−1^ (Table 2).

**Table 2:** Matrix of twenty mixtures of hydrolysates used for supplementation using a simplex lattice design

Mix #	Ratio of additional hydrolysates (%)*			

	Trypton N1	Casein peptone plus	Soy peptone E110	Soy peptone A2SC
1	100	0	0	0
2	0	100	0	0
3	0	0	100	0
4	0	0	0	100
5	50	50	0	0
6	50	0	50	0
7	50	0	0	50
8	0	50	50	0
9	0	0	50	50
10	33.3	33.3	33.3	0
11	33.3	0	33.3	33.3
12	0	33.3	33.3	33.3
13	33.3	33.3	0	33.3
14	17	17	67	0
15	0	67	17	17
16	67	17	17	0
17	70	10	10	10
18	10	70	10	10
19	10	10	70	10
20	25	25	25	25
SFM	-	-	-	-

* The total amount of hydrolysates supplemented was always 5&2 g/L, equal to 100%

Productivity and viability responses were measured for each combination (Tables 3 and 4). Comparison of the results of peptone supplementation with a non-supplemented medium (negative control) was performed in triplicate in parallel tests[3,14]. Results of productivity and growth in response to hydrolysate additives were evaluated using the analysis of variance (ANOVA). The specific productivities with *P* value <0.05 were considered statistically as significant responses. The optimal ratios of the four hydrolysates were predicted using achieved plots aiming at maximum specific productivity, and 15 combinations with higher responses were selected (Table 5).

**Table 3:** Twenty peptone mixtures with 2g/L in SFM and its effect on μ and q

Mix#no	MAX cell density	μ ±SD	Max.protein production	qPrptein (unit/cell/day)
1	642,500	0.53525±0.049	9.833855799	1.29679E-05
2	712,500	0.635231±0.089	10.43469175	1.29335E-05
3	517,500	0.475346±0.086	10.17345873	1.52312E-05
4	412,500	0.361959±0.024	9.332288401	1.5896E-05
5	590,000	0.540903±0.035	9.823406479	1.36244E-05
6	637,500	0.579618±0.137	9.206896552	1.21977E-05
7	522,500	0.463121±0.002	9.698014629	1.44389E-05
8	505,000	0.463121±0.134	8.104493208	1.23061E-05
9	687,500	0.617372±0.038	8.935214211	1.13156E-05
10	667,500	0.602611±0.007	8.303030303	1.07027E-05
11	787,500	0.685273±0.060	8.726227795	1.01785E-05
12	742,500	0.655853±0.011	9.269592476	1.12064E-05
13	582,500	0.534506±0.051	9.656217346	1.34936E-05
14	632,500	0.575681±0.008	8.600835946	1.14482E-05
15	485,000	0.442916±0.006	9.227795193	1.43408E-05
16	625,000	0.569717±0.056	8.684430512	1.16416E-05
17	635,000	0.577654±0.033	8.950888192	1.18862E-05
18	695,000	0.622797±0.128	8.496342738	1.06899E-05
19	677,500	0.610046±0.054	9.499477534	1.21364E-05
20	540,000	0.496626±0.06	9.394984326	1.37229E-05

**Table 4:** Twenty peptone mixtures with 5g/L in SFM and its effect on μ and q

Mix#no	MAX cell density	μ ±SD	Max.protein production	qPrptein(unit/cell/day)
1	585000	0.536647±0.030	8.774834	1.22E-05
2	507000	0.465097±0.024	10.72848	1.63E-05
3	670000	0.60448±0.026	7.086093	9.11E-06
4	620000	0.565701±0.005	11.02649	1.49E-05
5	602000	0.626381±0.070	10.72848	1.67E-05
6	592000	0.542595±0.038	7.516556	1.04E-05
7	730000	0.647364±0.029	7.218543	8.82E-06
8	562000	0.516592±0.003	7.18543	1.03E-05
9	645000	0.585466±0.050	8.741722	1.48E-05
10	770000	0.674037±0.036	7.483444	8.85E-06
11	565000	0.519254±0.012	8.476821	1.21E-05
12	650000	0.589327±0.043	5.89404	7.71893E.06
13	635000	0.577654±0.039	6.688742	8.88E-06
14	630000	0.573701±0.022	7.880795	1.05E-05
15	727000	0.645305±0.021	7.549669	9.24E-06
16	752000	0.662209±0.035	7.317881	8.78E-06
17	880000	0.740802±0.016	6.788079	7.40E-06
18	830000	0.71554±0.025	7.251656	8.24E-06
19	997000	0.503066±0.029	7.582781	1.10E-05
20	932000	0.803217±0.012	6.953642	7.01E-06

**Table 5:** The matrix of DOE predicted peptone mixtures and its effect on *µ* and *q* based on *in vitro* experiments

Media/Mix #	Tryptone N1from casein	Casein peptone E1	Soy peptone E110	Soy peptone A2SC	Amount of peptones mixture (g/L)	MAX.pro.production (miliunit/ml)	µ (/day)	q (micro unit/cell/day)
SFM	0	0	0	0	0	5039.06	0.84±0.001	4.83±0.006
1	0	0	27.08	72.92	5	4982.91	0.73±0.038	5.45±0.202
2	31.27	68.73	0	0	5	4836.43	0.64±0.021	5.92±0.141
3	63.09	36.91	0	0	5	4912.11	0.64±0.009	5.99±0.063
4	73.88	26.12	0	0	5	4921.88	0.71±0.033	5.51±0.183
5	81.92	18.08	0	0	5	4948.73	0.68±0.009	5.81±0.054
6	0	0	24.01	75.99	5	4809.57	0.63±0.014	5.95±0.097
7	38.95	61.05	0	0	5	4780.27	0.79±0.04	5.63±0.445
8	48.89	46.14	4.97	0	5	5146.48	0.66±0.014	6.18±0.094
9	75.52	24.48	0	0	5	4995.12	0.66±0.009	6±0.060
10	33.5	0	27	39.5	5	5085.45	0.64±0.031	6.21±0.213
11	8.76	0	0	91.24	2	4958.50	0.67±0.025	5.83±0.152
12	48.44	50.31	0.44	0.81	2	5202.64	0.68±0.013	6.1±0.086
13	67.88	31.49	0.26	0.36	2	5112.30	0.72±0.012	5.69±0.069
14	61.23	36.91	0.72	1.14	2	4985.35	0.74±0.278	5.39±0.142
15	21.16	0	0.01	78.83	2	5002.44	0.63±0.029	6.18±0.203

Values are means±SD of three independent experiments

### Protein expression analysis

Regarding the previous studies[10,11], expression level of truncated-mutant t-PA concentration in the culture medium was determined using ELISA based on amidolytic activity method on day 10. The amidolytic activity test (Biopool), known as Chromolize t-PA Assay Kit, is a biofunctional immunosorbent assay based on capturing t-PA by sp-322 monoclonal antibodies coated on the microtest wells. After fulfilling the steps from the kit’s manual, the absorbance of each sample was read at 405 nm and 492 nm by spectrophotometer. Absorbance at 492 nm was measured and subtracted from 405 nm. Various dilutions of each sample were assayed. The amount of developed color was proportional to the amount of t-PA activity in the sample.

### Cell viability evaluation

**Fig. 1:**
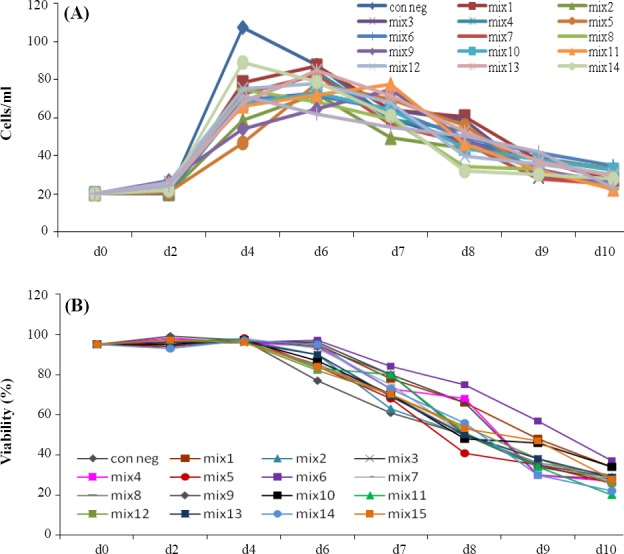
Cell density (A) and viability (B) results for t-PA producing rCHO-DG44 cells in a 10-day batch culture supplemented with 15 peptone combinations predicted by DOE. d, day

Viable cell concentration and viability were determined during cultivation period (Fig.1B). Cell density and viability were assessed by the Trypan blue dye exclusion method (1:1 mixture of 0.2% Trypan blue in normal saline and cell samples) using a haemocytometer (Neubauer improved, Brand).

### Evaluation of specific growth rate and specific productivity

The specific growth rate (*μ*) value was calculated by plotting the logarithm of viable cell concentration versus culture time during the exponential growth phase. The specific productivity (q) value was evaluated from a plot of the antibody concentration against the time integral values of the viable cell growth curve.

### Cell lysis and total protein extraction for proteomics analysis

To quantify cellular protein content, cells were harvested on day 10 of culture at late stationary phase when the number of cells was 1×10^7^, and then the cells were washed three times with ice-cold wash buffer (Tris 3 mM, sucrose 250 mM). A total of 10^7^ cells from each sample were lysed for 30 min in 450 µL lysis buffer (7 M urea, 2 M Thiourea, 40 mM Tris, 4% CHAPS, 0.2% [w/v] Bio-Lyte 4/7 ampholyte] Bio-Rad Laboratories, Hercules, CA), and 50 mM DTT]) at 4ºC. After centrifugation at 14,000 ×g at 4ºC for 10 min and the centrifugation at 14,000 ×g at 4ºC for 10 min, the supernatant was collected and kept at -20ºC for further analysis. Protein concentration was determined using the Bradford method.

### Two-dimensional gel electrophoresis

Samples (1 mg) were loaded onto the 17 cm IPG strips (pH 4-7, Bio-Rad Laboratories, USA) following mixing with rehydration buffer (8 M Urea, 4*%* CHAPS, 0.2% Biolyte 4/7, 0.0002% Bromophenol Blue, and 50 mM DTT). After rehydration for 16 hours, isoelectric focusing was performed at 20ºC for a total of 50 kVh using the PROTEAN® IEF Cell System (Bio-Rad Laboratories). Prior to SDS-PAGE, the strips were equilibrated for 20 min in equilibration buffer 1 (6 mM urea, 2% SDS, 20% glycerol, 0.05 M Tris-HCl, pH 8.8, and 2% DTT), followed by equilibration for 20 min in equilibration buffer 2 (6 mM urea, 2% SDS, 20% glycerol, 0.05 M Tris-HCl, pH 8.8, and 2.5% iodoacetamide). IPG strips were then placed on top of 12% SDS-polyacrylamide gels (18 cm×20 cm×1.0 mm) and embedded with 1% agarose. Electrophoresis was carried out at two steps, 16 mA for 30 min, followed by 24 mA for 5 hours, using PROTEAN^®^ II XL Cells (Bio-Rad -USA). Gels were then stained with modified colloidal Coomassie Blue[15].

### Image analysis

Gels were scanned at a resolution of 300 dpi using a GS-800 calibrated densitometer (Bio-Rad Laboratories). The gel images were analyzed by Image Master 2D Platinum 6.0 software (GE Healthcare, Sweden). The software was used to automate the process of detecting and matching protein spots between the images. The significant expression changes (*P*<0.05) were determined using statistical tests such as student’s *t*-test on % volume of matched spots between the groups. Among the selected spots, fold change >1.3 and coefficient of variation <20 were considered for each spot.

### In-gel digestion and protein identification by MALDI-TOF/TOF

The selected spots were manually excised from two dimensional electrophoresis (2-DE) gels and dried completely. The gel pieces were washed in 100 mM ammonium carbonate at the room temperature for 1 hour, followed by a second wash in 50% acetonitrile/100 mM ammonium bicarbonate. The gel pieces were washed again with 50% acetonitrile/100 mM ammonium bicarbonate and then dehydrated by incubation with 0.1 ml acetonitrile at room temperature for 10 min. The gel pieces were dried and then resuspended in freshly prepared trypsin solution (0.5 mg modified porcine trypsin in 25 µl 20 mM ammonium bicarbonate) and later incubated at 37ºC for 240 min. The peptides were extracted from the gel pieces by soaking twice in 0.1 ml of 50% acetonitrile, 0.1% trifluoroacetic acid and transferred into a 96-well plate to dry. The tryptic peptides were resuspended in 3 µl of 50% acetonitrile and 0.1% trifluoroacetic acid. The resuspended tryptic peptides (0.3 ml) were spotted onto a steel Applied Biosystems 192 sample MALDI target plate, and mixed (while wet) with 0.3 ml of 90% saturated alpha-Cyano-4-hydroxycinnamic acid in 50% acetonitrile and 0.1% trifluoroacetic acid. The dried samples were analyzed using a tandem mass spectrometry (4700 Proteomics Analyzers, Applied Biosystems, Foster City, CA). Each sample was calibrated internally by the reference to specific autolytic fragments of trypsin. The peptide mass fingerprint and tandem mass spectrometry information were searched automatically against the National Center for Biotechnology Information (NCBI) *Cricetulus griseus* database using the MASCOT search engine (Matrix Science). For each search, the mass tolerance for parent ions and fragment ions were set at 100 ppm and 0.5 Da, respectively. The search settings allowed one missed cleavage with trypsin and two modifications (carboxamidomethylation of cysteine and oxidation of methionine). The statistical confidence limits of 95% were applied for protein identifications.

### Western blotting

Protein extracts (30 μg) from control, Mix. #8, Mix. #10, and Mix. #12 groups, were loaded on 12% SDS polyacrylamide gel. After SDS-PAGE, proteins from gels were electrotransfered in a Semi-Dry Trans-Blot Cell (Bio-Rad- USA) to a nitrocellulose membrane (Hybind ECL, GE healthcare, UK) using transfer buffer (25 mM Tris, 192 mM glycine, and 20% methanol). After an overnight incubation, in blocking buffer (2.5% skim milk, 2.5% glycerol, and 0.05% tween-20 in TBS) at 4ºC, the membrane was washed three times in TTBS (100 mM Tris–HCl, 0.9% NaCl, and 0.05% Tween-20, pH 7.5) for 10 min. It was then incubated for two hours in blocking buffer containing polyclonal antibodies against vimentin (1:5000, Abcam, UK), ferritin heavy chain (1:1000, Zist Fannavaran Sina, Iran), pyruvate dehydrogenase (1:25,000, GeneTex, UK), and monoclonal antibodies against N-myc downstream-regulated gene 1(NDRG1) (1:1000, Abcam, UK) and β-actin as a control protein (1 µg/ml, Sigma-Aldrich, UK). The membrane was then incubated with peroxidase-conjugated anti-mouse and anti-rabbit IgG as secondary antibodies (1:1000, Razi Biotech, Iran). Subsequently, the immunoreactive bands were detected by ECL plus kit (GE healthcare, USA) using Kodak Image Station 4000MM Pro.

## RESULTS

Among the studied peptones, Casein peptone plus, Tryptone N1 from casein, Soy peptone A2 SC, and Soy peptone E110 had the most positive effect on specific growth rate (µ) and specific productivity (q) values[3]. Therefore, recombinant CHO (rCHO)-DG44 cells were cultivated in basal SFM supplemented with different combinations of these four hydrolysates (Table 2).

For each hydrolysate composition, viable cell and product concentration were determined during batch culture process. To determine the best ratio of hydrolasate mixtures that yield maximum specific productivity (q), the data obtained from Table 2 and related responses (Tables 3 and 4) were analyzed by DOE software Design-Expert software, and subsequently 15 different hydrolysate compositions were predicted.

The effect of each DOE-suggested mixture on µ and q were confirmed by performing a batch culture process with supplementing the hydrolysates mixture in basal SFM (Table 5). Figure 1 shows the results of cell density and viability from each of 15 predicted compositions of peptones by DOE.

As indicated in Table 5 and Figure 1, all predicted compositions showed an increase in specific productivity (q) along with a decrease in growth rate (µ) compared to the basal SFM. Among all fifteen conditions, four peptone compositions showed better results for specific productivity (compositions #8, 10, 12, 15).

### Proteome analysis by two dimensional

**Fig. 2:**
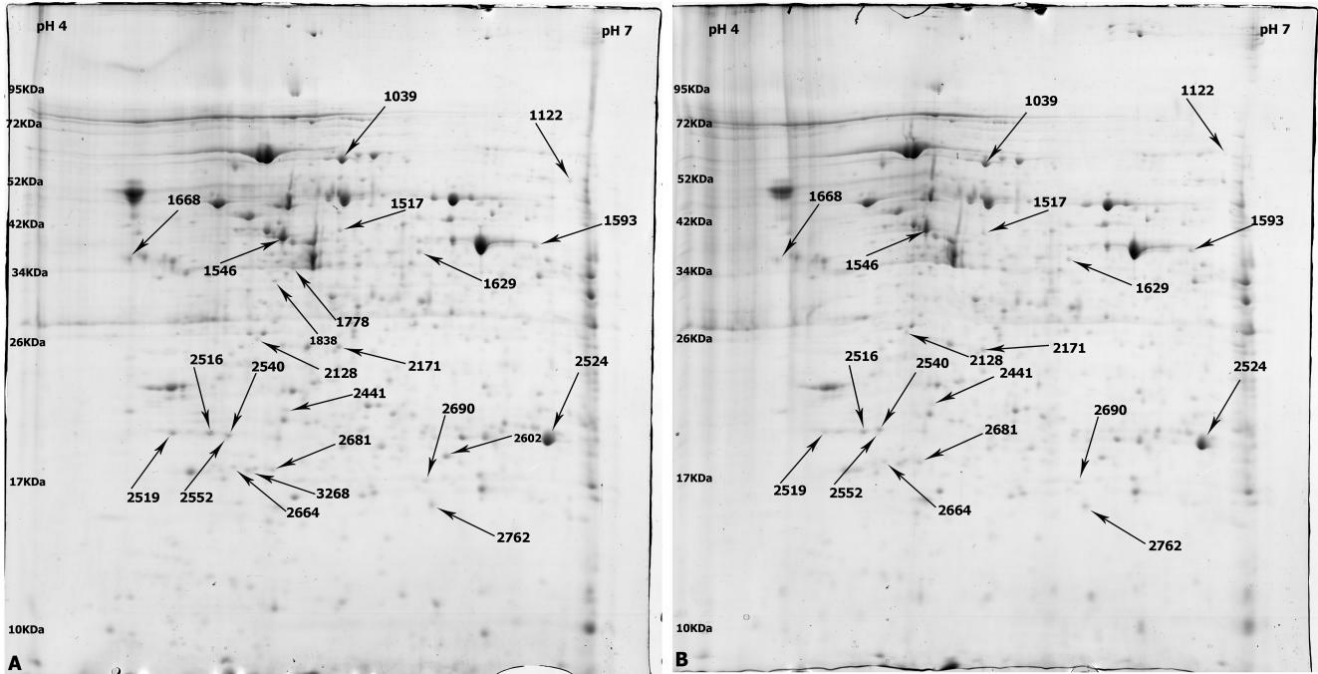
The representative two-dimensional gel electrophoresis images of rCHO-DG44 cultivated in basal SMF as negative control (A) and supplemented with Mix Hydrolysate 8 (B). Differentially expressed protein spots were indicated by arrows.

Two-dimensional electrophoresis on proteins extracted from rCHO-DG44 cells, which were treated with hydrolysate Mix. #8, 10, and 12 in parallel with the control group cultivated in basal SFM, was performed by loading 1 mg of each lysates onto 17 cm IPG strips (pH 4-7). The protein spots were visualized with colloidal Coomassie Blue staining (Fig. 2), followed by analyzing with Image Master 2D Platinum Software (version 6).

The protein profiles of three experimental replicates of each treated group were compared with three replicates of control group individually. To determine the significant changes in protein expression in Mix. #8, 10, and 12 compared to negative control group, statistical tests such as student’s *t*-test with *p* value <0.05 on vol. % on matched spots were performed.

The protein profile of rCHO-DG44 cells in basal SFM supplemented with Mix. #8, 10, and 12 represented 23, 11, and 7 differentially expressed protein spots, respectively, as compared to the control cultures. Among 23 differentially expressed proteins in Mix. #8, 13 spots were up-regulated, and 10 spots were down-regulated. In case of using Mix. #10, 9 spots were up-regulated, and 2 spots were down-regulated. Among 7 differentially expressed proteins in Mix. #12, only 1 spot was up-regulated, while 6 were down-regulated. Totally, 41 protein spots with significant expressional changes were detected among these analyzed groups, which their fold change was more than 1.3.

**Fig. 3:**
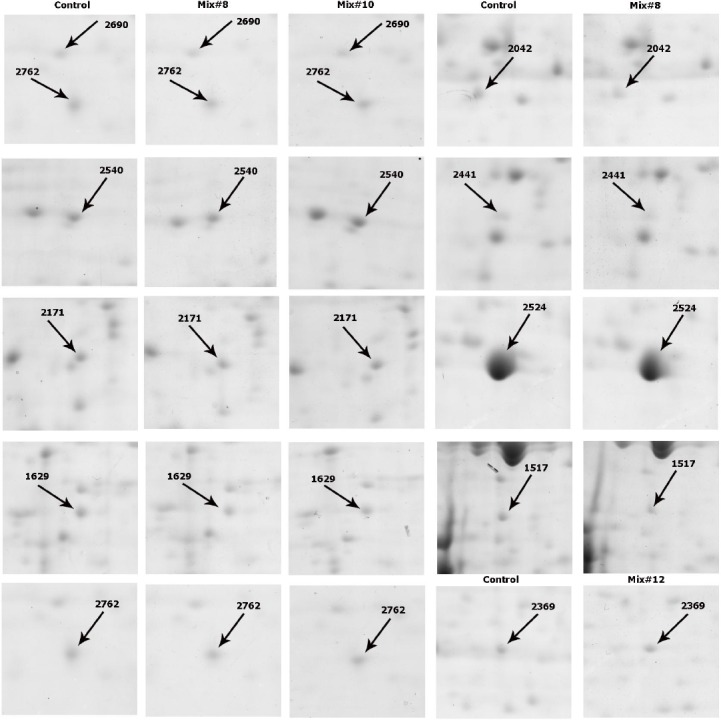
Two dimensional partial images of some differentially expressed protein spots. left panels indicate the gel images of rCHO-DG44 in SFM, and the right panel indicates the gel images of rCHO-DG44 cultivated in Mix. #8, Mix. #10 and Mix. #12.

Figure 3 shows the images of the representative spots with significant expressional changes. Among these protein spots, 11 spots were identified successfully through MALDI-TOF/TOF analysis and MASCOT searching against *Cricetulus griseus* database (Table 6).

**Table 6:** The list of identified differentially expressed protein spots in rCHO cells supplemented with mix. # 8, 10, compared to control group by MALDI-TOF/TOF

Spot no.^a^	Protein Name	Accession no.^b^	Theoretical Mr/pI	Protein score	Peptide count	Sequence coverage (%)	Fold change Mix 8/C	Fold change Mix 10/C
2540	Vimentin	gi|860908	44611/4.75	416	6	16	1.7	2
1629	NDRG1	gi|344259130	35603/5.89	106	1	4	1.5	1.8
2171	pyruvate dehydrogenase E1	gi|625199086	39732/6.41	280	5	18	1.4	1.5
2042	60S acidic ribosomal protein P0 isoform X2	gi|344237054	30039/8.68	339	5	27	0.6	-
2441	Proteasome subunit alpha type-3	gi|344250391	29667/6.38	132	2	8	0.6	-
2762	ferritin heavy chain	gi|625224189	21644/5.74	323	4	30	1.5	1.5
2524	triosephosphate isomerase isoform X1	gi|625183009	32313/5.36	487	5	23	0.7	-
2602	thioredoxin-dependent peroxide reductase	gi|354476011	28336/6.79	174	2	17	-	1.4
1517	Gluthation synthetase-like	gi|537151483	53458/5.4	372	4	13	0.6	-
2690	Cytidine monophosphate (UMP-CMP) kinase 1	gi|17389257	26125/8.13	134	2	14	1.4	1.3

a Spot numbers belonged to the identified spots in 2DE gels indicated in Figure 2;

b protein accession numbers were obtained from *Cricetulus griseus* using MASCOT peptide Mass Fingerprint peptide software.

**Fig. 4:**
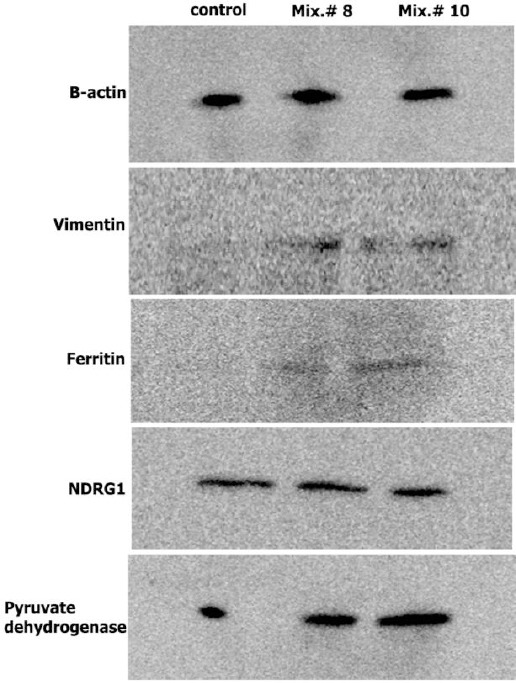
Immunoblot analysis. Immunoblot analysis of ferritin heavy chain, vimentin, and NDRG1 and pyruvate dehydrogenase in rCHO-DG44 cells in basal SFM as control and supplemented with Mix. #8 and Mix. #10. β-actin was used as reference protein.

Five spots that were detected in both Mix. #8 and 10 represented similar expressional changes, and that all were up-regulated compared with control group. These spots were identified as vimentin, NDRG1, pyruvate dehydrogenase E1 (PDHE1), Uridine monophosphate/Cytidine monophosphate (UMP-CMP) kinase 1, and ferritin heavy chain. Among protein spots with significant expression changes in group Mix. #12, only one spot was identified as alpha-enolase (data is not mentioned in Table 6). The identified proteins belonged to cytoskletal, growth and differentiation proteins, glucose metabolism, and iron hemostasis pathways. Among identified proteins, the expression of four proteins, including vimentin, NDRG1, PDHE1 beta subunit, and ferritin heavy chain were confirmed by immunoblotting analysis. Anti-β-actin antibody was used as the reference protein (Fig. 4).

## DISCUSSION

Recombinant protein production in mammalian cells is affected by culture conditions and nutrient supplementation. In this study, we took advantage of DOE to improve specific productivity of t-PA in CHO-DG44 cells through media supplementation with different mixtures of plant- and animal-derived hydrolysates.

To understand the intracellular responses to peptone supplementation and subsequent high yield of specific productivity, the profiles of two-dimensional gel electrophoresis of CHO cells cultured in SFM were compared with those of cells cultured in peptone-enriched SFM. Twelve spots with significant expressional changes were selected, and 11 distinct proteins were identified by MALDI-TOF/TOF mass spectrometry. Based on gene ontology terms of biological process, identified proteins were categorized into three groups: those involved in growth and differentiation, structural proteins, and proteins with metabolic roles[16]. Of 5 proteins, 11 showed similar expression patterns (up/down regulation) in Mix. #8 and 10, which can be appropriate candidates for future studies.

Type III intermediate filament, vimentin, has been reported in several proteomics analysis of mammalian cells. Kim *et al*.[8] determined the up-regulation of vimentin in CHO cells cultured in hydrolysate supplemented SFM. Rat and human fibroblast cells, cultured for one week in batch mode, were fed with carnosine, a dipeptide of beta-alanine and histidine, and its related dipeptides expressed high level of vimentin[17]. The antioxidant effect of carnosine that inhibits reactive oxygen species (ROS) signaling pathway was supposed to increase the expression of vimentin and reduce growth rate[17]. The antioxidant effect of peptones, especially soy peptones has been proved[18-20] and inhibit cellular responses mediated by ROS and cause the elevated level of vimentin. Also, vimentin down-regulation has been reported in cells facing transient oxidative stress[21]. The antioxidant effect of peroxiredoxin III, discussed later, might have emphasized this up-regulation.

Gene-encoding protein NDRG1 is a P53 target gene and plays roles in differentiation and tumor suppression[22]. In a similar study in which the SFM was enriched with hydrolysates, the expression of NDRG1 in day 4 of culture was decreased[8]. On day 4, the cells were at growth phase, and down-regulation of NDRG1 for cells with higher growth rate was expected. In our study, the sampling was on day 10, and cells were at late stationary phase. Also, the cells with higher specific productivity had lower population in time of sampling compared to control group, which explains the higher expression of NDRG1.

Ferritin is the major protein in storage and hemostasis of iron through ferroxidase activity. Ferritin heavy chains and ferritin light chains assemble to form cytosolic ferritin as a 24-subunit protein with variable ratio of two subunits[23]. Since the ferritin heavy chain contributes to the catalytic and antioxidant activity of ferritin, its elevated level in this study suggests higher antioxidant activity in hydrolysate-supplemented media. Diverse expression (up-/down-regulation) of ferritin has been reported in several experiments[21,24,25]. Prentice *et al*.[26] reported that the level of ferritin heavy chain expression elevated in late stationary phase, which is in correlation with the higher expression of ferritin in the late stationary phase of our experiment.

UMP-CMP kinase phosphorylates CMP and UMP as well as dCMP and dUMP to corresponding nucleoside diphosphates in expense of ATP. Both *de novo* and salvage pathways of nucleotide synthesis depend on UMP/CMP kinase for conversion of nucleotide monophosphate to nucleotide diphosphate[27]. Up-regulation of this protein leads to higher metabolic activity and protein synthesis that could be effective on increasing protein production in hydrolysate-supplemented CHO cells.

PDHE1 is a member of pyruvate dehydrogenase complex that is located in mitochondrial matrix and catalyzes the conversion of pyruvate to the acetyl-CoA. The limiting reaction of this pathway is the one catalyzed by PDHE1. Regulation of the activity of this complex is also controlled with phosphorylation and dephosphorylation of PDHE1[28]. The up-regulation of beta subunit of this enzyme in our study may reflect the higher metabolic activity of cells and energy production through citric acid cycle in mitochondria. The increased activity of mitochondria is also expected as a result of higher expression of peroxiredoxin III, discussed later. The increase in metabolic activity and energy production has been reported for other enzymes of glycolysis and citric acid cycle in peptone-supplemented CHO cells[8].

Peroxiredoxin-3 is the mitochondria-located isoform of peroxiredoxin family that catalyzes the reduction of ROS. An important target for this protein is H_2_O_2_, which induces oxidative damage and apoptosis. Several studies have shown the role of this thioredoxin-dependent enzyme in cell proliferation through the NFk-B signaling pathway[29] and inhibition of apoptosis[30]. Kim *et al*.[8]indicated the up-regulation of peroxiredoxin-6 and down-regulation of peroxiredoxin-2 in CHO cells cultivated in SFM supplemented with hydrolysates. The overexpression of peroxiredoxin-3 in mitochondria contributes to the elimination of H_2_O_2_ produced through respiratory chain[31] and provides an opportunity for cells to increase their metabolic activity and express the recombinant t-PA without facing negative effects of ROS. Peroxiredoxin-3 activates NFk-b that is involved in ferritin heavy chain gene expression[21]. This interaction explains coincident up-regulation of ferritin and peroxiredoxin III for cells harvested on day 10.

Among detected proteins, GSH synthetase-like, 60s acidic ribosomal protein p0, proteasome subunit alpha type 3, and triose phosphate isomerase were observed only in culture medium supplemented with Mix. #8, and all were down-regulated.

The existence of glutathione synthetase-like protein has not been proved yet[32], and there is not enough data about its function. Alignment of its amino acid sequence with protein sequence of glutathione synthetase results in more than 90% sequence similarity; hence, we considered the roles of glutathione synthetase-like similar to that of glutathione synthetase. Glutathione synthetase protein catalyses the last stage of glutathione biosynthesis[33]. Glutathione is an important antioxidant and also a regulator of DNA and protein synthesis[33]. Triose phosphate isomerase is involved in glycolysis and catalyzes the interconversion of glyceraldehyde 3-phosphate and dihydroxyacetone phosphate[34]. 60S acidic ribosomal protein is a component of 60S ribosomal subunit and is functionally equivalent to the *E. coli* L10 ribosomal protein[35].

Proteasome subunit alpha type-3 participates in growth and proliferation pathways. This proteasome degrades target proteins in an ubiquitin-dependent manner. Two of the target proteins are p21WAF1/CIP1[36] and retinoblastoma, promoted by MDM2[37], which suggests the role of this proteasome in positive regulation of cell cycle. Kim *et al*.[8] reported the up-regulation of proteasome subunit alpha type-5 in CHO cells with high growth rate.

The major difference of mix #12-enriched SFM with mix #8- and #10-enriched SFM is the final concentration of peptone, 2 g/L in mix #12, and 5 g/L in mix #8 & #10. However, the specific protein productivity is similar for all three mixes. The only detected protein for this composition was alpha enolase-1.

ENO-1 is a metabolic enzyme and catalyzes the reversible dehydration of 1, 3-bisphosphoglycerate into phosphoenolepyruvate[38]. Up-regulation of α-enolase and other glycolysis proteins, pyruvate kinase and phosphoglycerate kinase, has been noticed in CHO cells with higher specific recombinant protein production[8,39].

Proteomics analysis of CHO cells cultivated in peptone-enriched SFM provides valuable data on molecular mechanisms of protein production. Results of proteomics analysis revealed the roles of proteins involved in energy metabolism pathways. Proteins of glycolysis and cell hemostasis allow high energy production and metabolic activity. Up-regulation of two mitochondrial proteins, PDHE1 and peroxiredoxin-3, may emphasize the higher metabolic activity.

Based on antioxidant effect of peptones and also the functions and characteristics of discussed proteins, vimentin, peroxiredoxin III, and ferritin heavy chain, prevention of oxidative stress seems to be an effective factor in improving culture condition and recombinant protein production. Further studies with proteomics approach are needed to provide new insights into the molecular mechanisms involved in recombinant protein production and cell engineering for higher specific productivity.
